# Cholera—the Sedative Plan of Treatment

**Published:** 1849-09

**Authors:** I. L. Adkins

**Affiliations:** Philadelphia; Physician to the Moyamensing Cholera Hospital


*Cholera—The Sedative plan of Treatment.* By I. L. ADKINS, M. D., Philadelphia; Physician to the Moyamensing Cholera Hospital. (Communicated in a letter to Prof. Dunglison.)

In sketching, at your request, and for your perusal, a few cases of Asiatic Cholera, with the treatment, I hope I shall not be understood as claiming any particular merit or superiority for the plan pursued and found successful at the Moyamensing Hospital; although, without my knowledge or consent, and to my annoyance, comparisons have been made, and my name paraded in the public prints, as certifying to a statement, which, however true, was given for a strictly private purpose, as distinctly understood by a number of persons present at the time.

So also I can say of what purported to be *a certificate* from my friend and colleague, Dr. Smith.

My communication will be mainly in reference to the point on which all are most interested, the *treatment* of Epidemic Cholera. As to the cause, pathology, &c., more curious than important, and less understood than either, I leave them to others, abler and more disposed than I am to discuss them.

In the beginning of the epidemic, and occasionally since, I employed the various agents and modes of treatment set forth by different writers, certain of them claiming to be almost specific. If I had ever believed in such a thing as a specific in disease, I should, before this, have been driven to abandon the belief, by the protean character of the disease in question, and the resources necessary to be drawn upon to meet the various indications as they have presented themselves.

You are aware that I have, from the first, whenever I have had it in my power, employed what I call the *sedative plan of treatment*, giving it preference over the strictly stimulant, which has been most generally, perhaps, in favor. Let it be understood here, that it is at the outset, the invasion of the disease, when the morbific influence, (whatever it may be,) the "cholera poison," is doing early, its fatal work, that this treatment is most serviceable. At any time, however, from the beginning of the attack to approaching collapse, and even occasionally in collapse, the destructive condition may be removed by it, and the patient saved from death. At this stage of the disease, when the cause may have ceased to be in operation, and the *effects* have to be combated, stimulants and tonics are judiciously employed.

Impressed with the idea, that there is, in cholera, irregularity in the organic actions, the brain being at times greatly excited ; at others, the alimentary tract, and at others, the muscles alone ; with, in almost all cases, the escape of fluids from the lining membrane of the stomach and bowels, the result of organic excitement, I was led to adopt, almost exclusively, the *sedative plan* of treatment, which has, in the end, proved successful and satisfactory. The agents employed were opium and blood-letting, assisted by warm applications and warm drinks, until diaphoresis was fairly established, combined with perfect rest in the horizontal posture. These means, as all know, are well adapted to many conditions which we meet with, not only in cholera, but in other diseases ; to relax spasm ; to equalize the circulatory movements ; allay excitement ; produce sleep and warm perspiration, and check (opium especially) the inordinate discharges, whether rice-water or other. These are the means, singly or conjoined, employed with judgment and discrimination, which will, I believe, in most cases be found all-sufficient.

I will commence with a case which occurred to me in private practice before the hospitals were opened. I was called to see a daughter of Mr. W., in the district of Moyamensing. When I arrived, however, she was so far gone, that I did nothing in her case, especially as the physician in attendance was absent. She died, apparently in great suffering, in a short time, and before I had left the house. I learned that her mother had expired but a few hours before ; and while standing between these two victims of cholera, a third, the oldest daughter, a girl of about sixteen, was attacked just as I was taking leave. She threw from the stomach, without effort, two or three times in succession, the characteristic rice-water, and on inquiring I was informed that she had diarrhœa. Her countenance assumed the anxious expression, and her eyes the sunken appearance, with the blue ring around them, which is almost pathognomonic of this disease. Her face was bedewed with a cold sweat, and the pulse was becoming more feeble every moment. She quietly and sweetly remarked, that she

"would soon feel better;" but her mother and sister, who a few hours before were in tolerable health, were now lying dead before her. Her frantic father insisted that I should bleed her; that if I did not she would follow the other two, both of whom *he* had desired to be bled. Doubtless, the physicians arrived too late to do good by bleeding or any other remedies.

In this case I was on the spot in time. I saw the deadly stroke and felt my responsibility. I embraced the moment, boldly directed my agents, and the girl was soon well. From a vial of laudanum, which was on the table, I poured two teaspoonfuls nearly, and with a little water gave it to her at a draught. I tied up her arm, and took away about sixteen ounces of blood. A hot hot foot-bath was prepared, thick with mustard, in which her feet were kept 30 minutes, then taken out, wiped dry, and warm bricks applied, the body being well covered at the same time. A profuse perspiration broke out; the pulse came up from the moment the blood began to flow freely, and now was round and full. Directions were given in case of excessive reaction (which did not occur), and at my next visit, on the day following, I found my patient almost able, and quite anxious, to get up. This was my first case of Epidemic Cholera. Wine and water, and a little weak toddy were allowed her, and she was finally put on the sulphate of quinia.

She is now well.

Case 2. I was called, July 2d, to see Miss Q., æt. 14, residing in Seventh street near Wharton. She had the rice-water evacuations from the stomach and bowels, and was somewhat cramped. ℥xij. of blood were abstracted, and tinct. opii f.ʒj. given at one dose. Mustard was applied to the epigastrium, and warm applications to the feet. Very little else was done, and she awoke from a sound sleep, feeling pretty well. On the second day she was up; a tonic was ordered, and I saw her but once afterwards, on the third day, when she was quite well.

Case 3. Mrs. Q., mother of the last, æt. 35, was attacked July 6th with severe cramp and coldness of the extremities, intestinal spasm, and excessive vomiting. The ejections were at first clear as water, then like rice water, and finally tinged with bile. Bleeding, both general and local, was resorted to, for the purpose, in this case, of controlling the painful spasm and equalizing the circulation. Twenty ounces of blood were taken, and although in a semi-recumbent posture, and a very delicate looking woman, without the least appearance of syncope. Twenty leeches were applied to the abdomen, and, afterwards, a warm poultice for the internal pain and spasm. Tinct. opii. f.ʒj, with a little mint water was given at a draught. Under this treatment, the pulse, which was very small at the wrist, became full and soft, the pain and spasm were allayed, and she fell asleep. At my visit next day, she was found perfectly clear of cramp, and had no more vomiting. Wine and water, gruel, &c., were allowed, and she was ordered to remain perfectly still. Next day I was sent for in great haste, the messenger stating that all the symptoms had returned. She had gotten up against orders, and I found her vomiting, but without cramp. Ice, as before, was now given freely, and a sinapism applied over the stomach, but without much effect. The effervescing draught was given, which allayed the irritability, but for a time only; and neither pill, powder, nor mixture would remain. Morphia, camphor, creasote, brandy, all were alike rejected.

A blister to the epigastrium was ordered, the raw surface to be dressed with sulphate of morphia. A few drops of ol. succini in mucilage, which had also been directed, having given a little relief after I had gone, the attendants neglected the application of the cantharides, and early next morning I was summoned, and found matters as bad as before.

On consultation with a professional friend, whom I had asked to call with me, the emplast. cantharid. was applied, with cups and scarifications along the spine. An opiate was also given. I called again during the day and found her much improved.

Next day my friend called with me, and we were told that she was more unwell. The violent spasmodic action was more alarming to some of the friends than before; but it was not difficult to see that we had more of hysteria to contend with now than any thing else. Tinct. valerian. was ordered to be taken in drachm doses every hour, with occasionally liq. morph. sulph.; and an injection of assafœtida and tinct. opii was given. Gentle friction was kept up for a while, and she became quite composed. I had very little further trouble, except in dietetic management. The gastric irritability returned whenever food was taken; and for several days she took no nourishment but wine whey, and wine and water. Sago, farina, and broths, were finally tolerated; sulphate of quinia was given, and at the end of a week she was quite restored. There was no diarrhœa in this case, and I should have stated, that a purgative enema was given once or twice with the effect of checking the vomiting.

Case 4. I was called, July 17th, to see Mr. M., æt. 50, near Eighth and Carpenter. Found him going rapidly into collapse, almost pulseless, and with the peculiar whispering voice, blue and shrunken fingers, and a ghastly expression of countenance. He had suffered diarrhœa to continue for several days, and his legs were now very much cramped. He threw the rice water secretion from his stomach almost every minute while I sat by him. There was very little pulse at the wrist; but on auscultation I found the heart laboring violently.

I took about sixteen ounces of blood from his arm, and as it flowed at first with some difficulty, thick, and as black as pitch nearly, there was a sensible increase in the force of the pulse, and a diminution in its frequency. He expressed himself as feeling much relieved of the oppression and sense of suffocation at the præcordia. Laudanum being in the house, I gave him a large teaspoonful.

Mustard was applied to the epigastrium and a mustard foot-bath prepared. He fell asleep, and on awaking was found to be much better. Slight diarrhœa remaining, it was treated with

| | |
|---|---|
| Pil. hydrarg. | gr. xij. |
| Pulv. opii, | gr. iij. |
| Pulv. ipecac. | gr. ij. M. |

Made into twelve pills:—one every two hours.

Calomel, acetate of lead, and opium were afterwards given. He was doing very well, but from imprudently taking too much water, vomiting returned on the third day. Creasote gtt. iij., in aromatic mucilage, was ordered to be given occasionally, and ammon. carb. gr. v., every hour, when the stomach would bear it. The creasote was not taken, but the powders of carb. ammon. were,—to which was ascribed the cessation of vomiting when I saw him again.

He went on doing well, gradually regaining strength, and the shrivelled surface filling out again. I did not think to pay him another visit, and directed an infusion of prunus virginiana to be

50*

taken occasionally, with careful diet, the horizontal posture, &c. and cautioned him against drinking cold water. It seemed that the family had placed the vessel containing the infusion of the bark in a bucket of water at the side of the bed to be kept cool. In the night, according to his own statement, he helped himself largely to the water, and a fearful and rapidly prostrating discharge from the stomach and bowels was the consequence. I was not to be found in the night, and on calling in the morning, and seeing what had happened, I at first declined having anything more to do with the case, which was now hopeless. Stimulating and other means were resorted to, but without avail:—indeed the family had stimulated him to intoxication and nearly to death before I arrived. He died the next day, the sixth of his illness, a victim rather of imprudence than of cholera.

Case 5. George C., colored, æt. 30, was admitted into the Moyamensing Hospital laboring under symptoms of cholera, on the 4th of July. He had the rice-water evacuations and some cramp, and was treated with combinations of opium, camphor, and calomel, and soon appeared to improve. On the third day, however, he passed into a low adynamic condition, with muttering delirium. Blue mass and sulphate of quinia were given, and counter-irritation was employed on the epigastrium and spine. Wine and water and wine in gruel were allowed, and he was convalescent on the sixth or seventh day. Ptyalism was produced, which was the only further trouble.

Case 6. Samuel H., colored, æt. 13, was admitted into the Hospital on the 6th July. He had a cold, shrunken surface, scarcely any pulse at the wrist, cramps which caused him to scream constantly, and insatiable thirst. He had now no discharges, although he stated that he had had both vomiting and diarrhœa before coming into the house, which he attributed to eating pine-apples. He took camphor, chloroform, and opium, differently combined, and also small and repeated doses of calomel. These were discontinued, and he was allowed the free use of cold drinks, his only cry being for water, the bath, and fresh air. This was an interesting case in many respects. Pulseless as he was, he would suddenly rise from his bed, and start down stairs unassisted to get a bath, loudly screaming if opposed. Believing that he had gastro-enteric inflammation, and finding it impossible to control his desires, I

objected to anything like stimulants being given, and he was liberally allowed ice and ice water, and permitted to bathe. Without aid he would jump up and get into the bath, when not closely watched, and turn the water on himself. He seemed perfectly at ease in this condition, and would stealthily let his head sink far enough down for the water to flow into his mouth, as if he desired it by the gallon. On any attempt to remove him he would implore the nurses to let him remain and die in the bath. When taken out, he would roll to the nearest door and hang his head out to get the air. These scenes were enacted over and over again for two days and nights, during which he was watched as closely as possible, and his urgent wants to a great extent gratified. He died on the night of the 8th, his intellect unimpaired from first to last. A few hours before death, he became perfectly quiet and asked for nothing.

*Necroscopy.*—The stomach was nearly filled with pure pus, as tested by the microscope; and the intestines with pus, sanguineo-purulent matter and blood, in different parts of the tract. The mucous membrane was of a bright red color, continuously and in streaks and patches,—showing evidently that there had been intense inflammation. The colon was in an ecchymosed as well as inflamed condition. The valvulæ conniventes were greatly enlarged, and the glands of Peyer prominent and slightly ulcerated.

The few hours repose which he enjoyed before death was doubtless owing to the termination of the inflammation in suppuration. There was no congestion of the liver, lungs, spleen or brain. All, as also the heart and kidneys, were healthy-looking. When the aorta was cut, the heart emptied itself of very black, thick blood; no clot was found in the auricles or ventricles. The liver had been performing its functions, for the gall-bladder was distended with bile, and the duodenum contained a small portion, and was stained throughout its length, which may have been from transudation after death.

This was the most remarkable and unnatural case of nervous endowment—cold, pulseless, and dying, as the boy was, for 48 hours—that has ever come under my observation. Muscular contraction was noticed for some time after death.

Case 7. Mary Thedford, æt. 15, was brought into the house collapsed, July 15th. There was a very slight vibration at the wrist, and one or two physicians who were visiting us at the time proposed using the lancet. Veins of both arms and feet were opened, but no blood would flow from the orifices. Stimulants and cordials were given, and ice and ice water at her earnest entreaties. She died on the 17th, in about 48 hours from the time of attack.

Case 8. Eliza T., æt. 45, mother of the last described, was very suddenly attacked in the hospital while watching over her daughter. Almost without food or sleep, resisting all our efforts to separate her for a while from the precious charge over which she was incessantly bending, she fell down suddenly without any premonitions, to our knowledge, and went hurriedly into a fatal collapse, defying the most active and energetic measures. A vein of her arm was opened, and blood, which flowed only by forced means, as friction, &c., was taken to the amount of about 8 oz. Opium was freely given, and hot cordial drinks, warm applications and friction were employed; but all to no avail.

I have never seen two cases so parallel. Every want, entreaty, desire, expression and movement of the daughter was repeated by the mother; and before the former died, she constantly cried from one part of the house for her mother, while the mother in another part as often asked for the daughter.

Mother and daughter died in about 48 hours after the attack; the symptoms identical, but opposite treatment employed in the two cases. In neither were vomiting, diarrhœa, or cramps prominent symptoms. "Water, water, water," in a whispering, sepulchral voice, was the continual cry of both; and death in both cases seemed the result of a prostrating blow on the nervous system,—rapid exhaustion following without the usual drain from the stomach and bowels.

The orifices in the veins, in these and another fatal case, never closed, but remained gaping like a gash in the rind of an orange: there seemed not enough excitement in the system for the process of union.

I will here remark, that I have not been so well satisfied with blood-letting in hospital as in private practice. It is true we bled but few—not more than five or six cases—in two of which it failed. The constitutions of such as are brought to the hospital, are so broken down by depravity and destitution, that I concluded we were putting the remedy to a severe test, and have been perhaps over cautious on account of the two cases lost in which the lancet was used.

Case 9. Elizabeth K., æt. 40, Shippen street, was admitted July 24, with vomiting, diarrhœa, and cramps. Opium (gr. vj.) was given her; sinapisms were applied to the abdomen and ankles, and bottles of warm water about the body. The vomiting was very troublesome, as it has been in a majority of the cases brought to the Moyamensing Hospital,—the stomachs having been apparently injured by the miserable life of dissipation led by most of them.

This woman was soon better, but relapsed on the third day from drinking too much water, which she contrived to get. She must have had a hundred discharges both ways in two days following. Combinations of opium and plumb. acet., creasote, morphia, &c., were employed, but with no visible effect. She became completely exhausted, and was so far gone as to be able, by signs only, to beg that we would do no more for her. Impatient and dissatisfied with the result of the small and repeated doses employed, I gave her one night about 400 drops of laudanum, and introduced an opium pill of eight grains into the rectum: from that moment every unfavorable symptom disappeared, neither vomiting nor diarrhœa, which were immediatety arrested, appearing again for two weeks, for which time we were obliged to keep her, because she had no home. A blister was at the same time applied over the stomach, which probably had a good effect. She was supported for some time on porter, not being able to take any thing else. This was a remarkable case of recovery, and of tolerance of opium.

Case 10. Jane McC., æt. 26, was brought in, July 26th, quite feeble, but able to walk to her bed. Very little pulse at the wrist, and rice-water evacuations. That morning her husband was buried, having died of cholera the day before. Her child, which we suffered to be brought in with the mother, died next day in the hospital of inflammation of the brain.

Mrs. McC. took two pills containing six grains of opium; and subsequently tinct. opii. f.ʒj, with a little tinct. zingiberis. Bottles of hot water were placed about her person, and toast water was allowed to satisfy her thirst.

As free diaphoresis came on, toast water and thin rice flour water were freely given, with the effect of rapidly refilling the vessels, and filling out the shrunken fingers. The vomiting also ceased, and the stomach retained whatever was taken into it. Tonics and gentle stimulants were given,—as wine and water, porter, Huxham's tincture, &c. She was discharged well on the 30th.

Case 11. Ann L., was brought into the house in a fit of cholera and drunkenness combined. The cramps and spasms were most violent; no pulse at the wrist. Her face was livid from cerebral congestion. Veins in both arms were opened, but not a drop of blood would flow. I then opened the external jugular, and took about 16 oz. of blood, which was very dark and thick. The face became more natural in color; the circulation was restored to the capillaries of the extremities, and the pulse returned to the wrist. The patient was so far relieved that she went out of the house next day. The bleeding was followed by a draught of tincture of opium, and assafœtida.

Case 12. Joseph W., æt. 35, was admitted August 6th. His wife died of cholera at home the day before. He was in apparently hopeless collapse; pulseless, voiceless, and almost breathless. His eyes were sunk in the orbits; his fingers blue and shrivelled like a washerwoman's; tongue cold; the whole surface cold, blue, and bedewed with a death-like moisture. He looked like any thing else than a living man. Some physicians, who happened to be present when he was brought in, pronounced him moribund. The nurses, as usual, applied ten or twelve bottles of hot water, and hot bricks about him; a blanket was drawn closely round his neck, and two pills of eleven grains of solid opium were given him by my friend and colleague, Dr. Smith. He fell into a deep sleep, sweated freely, and awoke in a few hours expressing himself refreshed and feeling well. Toast water and cordial drinks were freely given, and the pulse gradually returned to the wrist. The usual stimulants and tonics were now directed, carbonate of ammonia, sulphate of quinia, wine and water, etc. He was gently rubbed with dry flannel several times a day, and was able to sit up a little on the second and third days. There was suppression of urine in this case, as in most of the bad cases of cholera we have had, for which mucilaginous drinks, as flaxseed tea, were freely given, with the desired effect. The patient was discharged well on the 14th. His cure seemed to some almost miraculous. I

must not omit to add, that be was pretty badly narcotised. This has not often occurred to us with our large doses of opium; but we are not alarmed if it does, as we are quite willing to replace the "cholera poison" by the "opium poison," if I may so speak, especially in extreme cases like this, and many others that have been brought to the Moyamensing hospital.

Case 13. Thomas B., colored, æt. 60, was brought into the house from Seventh and Baker, August 7th. He was placed opposite the case last described, and seemed about as bad, but not worse. The same general plan of treatment was carried out, but he died in a few hours after he was admitted. I have observed that, as elsewhere, cholera has proved more fatal in the colored than in the white cases under my charge.

Case 14. Patrick McGuire, æt. 36, was admitted August 7th, and was perhaps as far gone into collapse as cases 12 and 13. Tinct. opii. f℥ij. in camphor water was given him, and hot applications were employed with warm drinks and gentle friction for a short time. As the rice flour water and toast water and gruel were poured down during the sweating process, some one standing by dryly remarked, that he could see the *gruel running into his fingers*, so rapidly did they seem to fill up. A second dose was given,—tinct. opii, tinct. card. comp. āā f.ʒj., aquæ f℥j. Patrick raised his head up *in three hours*, and said, "thank God I feel nearly well." Wine and water, occasionally porter, and tinct. cinchon. comp. were given him, pro re natâ, and he was discharged well on the 16th of August.

Case 15. I was called late at night, July 16th, to see Mrs. A., in Carpenter street. She had severe cramps and vomiting of rice water. Her pulse was not so feeble as it was irregular. ℥xvj. of blood were drawn from her arm. She had some nervous excitement, and I ordered for her,—sp. æth. sulph. comp. f.℥j., morph. sulph. gr. i., to be taken in teaspoonful doses every hour. Mrs. A. was quite well in three days.

I have described as briefly as possible the history of a few cases of cholera, and the main treatment employed. They are but a few out of a great number, and are fair samples,—some of them mild in their character, others bad enough; all, however, here described, I believe to be true cases of epidemic cholera. More than a hundred have been under my charge in hospital and private practice; of all grades, from the most malignant to the mildest type. I often hear of gentlemen asserting, that there has been but little of real Asiatic cholera,—that they have seen no cases, &c. I only wonder that they, who doubt, have not taken the trouble to visit the hospitals and private cases in the Districts of Moyamensing and Southwark especially. They cannot account for so many cures, when it has generally been conceded that half of all attacked die. They say that all kinds of experiments have been made, and with similar results. I answer to this, that the more experiments we tried, and the more complex we made our prescriptions, the less successful were we in the Moyamensing hospital. Whereas, since our treatment has been simplified, and we have done boldly *and at once* what we intended to do, we have had cause to be gratified with the results. I look upon the disease as one that will not *wait* to be trifled with, and that a favorable or a fatal termination depends, in very many cases, on a slow, or a promptly vigorous plan of treatment. With this view, I very early decided to give all that I thought the patient would bear at a *single dose.* The agent I have preferred is opium in some form;—opium in decided, sedative doses. I am attached to this method of treatment for its *simplicity*, among other merits which I conceive it possesses. Many indications are met by opium. The inordinate discharges are arrested; spasm is relaxed; and irritability of stomach and general excitement allayed. Time is saved by the *one dose system;* the patient is left undisturbed to quiet and to sleep. Mr. Corbyn says "if there is a crisis in any disease, it is sleep in cholera." I think I have seen in many instances the truthfulness of this remark.

I have not entered fully into all the minutiæ of treatment,—the adjuvants, corrigents, &c. employed. My main object has been to show (whether considered philosophical or not) how we have combated and overcome *the cause of death;* not so much to detail the after treatment, without which the patient, in many instances, might get well. The symptoms have been watched, and met, as they appeared, on general principles.

Purgatives and purgative enemata have been occasionally employed with benefit;—anodyne injections, poultices, sinapisms, pediluvia and full baths, ice, ice water, carbonic acid water; the vegetable and mineral astringents by the mouth and rectum, to check the discharges, and occasionally, with benefit, injections of sulph. quinia in solution. Blisters, leeches and ice *externally*, and all kinds of friction, have been applied. Some of the agents afforded relief at one time, and failed at others.

As before remarked, we became tired of mixtures and combinations. We modified Hawthorne's, and all other plans—most of them being entirely too systematic and empirical for our use, and unnecessarily complex, seeing that the really essential, active ingredient of them all is opium. Opium, then, and blood-letting, to equalize the circulation, reduce irregular excitement, &c., judiciously employed, we regard as the Samsons in the cure of cholera; assisted, and materially, too, by warm applications, warm spiced drinks, strict quiet, and *horizontality*.

A formula, proposed by Prof. Meigs, I have employed with good effect at times, and often, in cases of irritable stomach, when nothing else was so well retained:

| | |
|---|---|
| ℞ Morph. Sulph. . . . . . . | gr. v. |
| Camphor. . . . . . . | gr. xx. |
| Ol. Cajeput. . . . . . . | gtt. x. |
| Tragacanth. | |
| Ext. Gentian. . . . . . . | āā q. s. |

M. To be made into a hundred pills.

I have sometimes added five grs. of piperine, in place of five grs. of the camphor, which to my taste makes the pills rather more agreeable. I have no great partiality for camphor or pepper; and not much more for calomel, in the *urgent stages*. We have used calomel and blue mass, but generally in mild cases when there was more of diarrhœa than any thing else, or in convalescence from the cholera attack. In some cases, under such circumstances, I have found benefit from mercurials, which have produced bilious evacuations, and removed offensive accumulations from the bowels, as often purgative enemata without mercury did. Diuretics were employed in many cases in which the urine was retained or suppressed. Frequently, it was necessary to draw off the water (which was always high colored) with the catheter.

I have said but little of post-mortem appearances. My time has been so much occupied in hospital and other duties, that, to my regret, I have made but few examinations. I have found, however, in those few, an inflamed and softened condition of the gastro-enteric mucous membrane, and in one case an eruptive, mottled appearance. I have hastily written out these homely, but I believe practical sketches. My object has not been to theorise or discuss mooted points, but, as before said, to offer you, not without distrust, an unvarnished statement of facts as I believe them to have occurred to me.

I should like to furnish you with a summary of the number of cases, deaths, and some other general points which might prove interesting, but have thought proper to withhold such a statement, till after some action shall have been taken on the Hospital reports by the Board of Health.

